# Uptake of *Clostridium botulinum* C3 Exoenzyme into Intact HT22 and J774A.1 Cells

**DOI:** 10.3390/toxins7020380

**Published:** 2015-02-02

**Authors:** Astrid Rohrbeck, Leonie von Elsner, Sandra Hagemann, Ingo Just

**Affiliations:** Institute of Toxicology, Hannover Medical School, Carl-Neuberg-Str. 1, Hannover D-30625, Germany; E-Mails: vonElsner.Leonie@mh-hannover.de (L.E.); hagemann.sandra@mh-hannover.de (S.H.); Just.ingo@mh-hannover.de (I.J.)

**Keywords:** C3 exoenzyme, *Clostridium botulinum*, vimentin, dynasore, endocytosis

## Abstract

The *Clostridium botulinum* C3 exoenzyme selectively ADP-ribosylates low molecular weight GTP-binding proteins RhoA, B and C. This covalent modification inhibits Rho signaling activity, resulting in distinct actin cytoskeleton changes. Although C3 exoenzyme has no binding, the translocation domain assures that C3 enters cells and acts intracellularly. C3 uptake is thought to occur due to the high concentration of the C3 enzyme. However, recent work indicates that C3 is selectively endocytosed, suggesting a specific endocytotic pathway, which is not yet understood. In this study, we show that the C3 exoenzyme binds to cell surfaces and is internalized in a time-dependent manner. We show that the intermediate filament, vimentin, is involved in C3 uptake, as indicated by the inhibition of C3 internalization by acrylamide, a known vimentin disruption agent. Inhibition of C3 internalization was not observed by chemical inhibitors, like bafilomycin A, methyl-β-cyclodextrin, nocodazole or latrunculin B. Furthermore, the internalization of C3 exoenzyme was markedly inhibited in dynasore-treated HT22 cells. Our results indicate that C3 internalization depends on vimentin and does not depend strictly on both clathrin and caveolae.

## 1. Introduction

C3 exoenzyme (C3) is produced by certain strains of different bacteria, such as *Clostridia*, *Bacilli* and *Staphylococci* [1,2,3,4]. C3 has been shown to be a Rho-ADP-ribosylating transferase, which inactivates the low molecular weight GTPases, RhoA, B and C, by transfer of the ADP-ribose moiety of the co-substrate NAD^+^ onto asparagine-41 of Rho [5,6]. The C3-mediated inactivation of Rho-GTPases causes the reorganization of the actin cytoskeleton [7], inhibition of proliferation and protection from cell death [8]. It has been shown that C3 induces neuronal growth of murine hippocampal neurons, which results in axonal and dendritic branching and growth [9].

C3 exoenzyme is a small monomeric basic protein (molecular mass of about 25 kDa, pI > 9) [10,11]. The crystal structure of C3 and structure-based sequence alignment resulted in the identification of the ARTT motif (ADP-ribosylating turn-turn motif), which is implicated in substrate recognition, but there are no hints about how binding to cells and uptake are mediated [11]. Despite the absence of a known receptor-binding domain, there is a lot of evidence suggesting that C3 is an intracellular-acting bacterial toxin. Recently, it was shown that C3 is taken up by different cultivated cells, as determined by ADP-ribosylation of RhoA, whereas the sensitivity towards C3 is very different [12]. It seems that primary neuronal cells are very sensitive to C3. Nanomolar concentrations of C3 lead to axonal and dendritic growth [13]. To date, the mechanism is not well understood by which C3 exoenzyme is internalized through the plasma membrane into the cytosol.

Several bacterial toxins specifically bind to target structures at cell surface and are endocytosed and transported to early endosomes [14,15,16]. Clathrin-mediated endocytosis is one of the most well-studied forms of membrane internalization. Within this pathway, dynamin mediates the pinching off of the budding coated vesicle from the membrane [17]. This endocytotic pathway is reported for diphtheria toxin [18,19]. In addition to this well-characterized endocytotic pathway, there are numerous clathrin-independent means of endocytosis. Caveolae-mediated endocytosis is one of the better characterized forms of clathrin-independent endocytosis [20]. Bacterial toxins, such as cholera toxin, are associated with caveolae and can be internalized by caveolae/raft-dependent endocytosis [21,22]. Moreover, clostridial binary toxins, such as *Clostridium botulinum* C2, use a clathrin-and caveolae-independent pathway, but this endocytosis is dependent on dynamin and Rho-GDI [23].

These observations suggest that many bacterial toxins use established endocytotic pathways for internalization into the target cells. However, little is known about the entry of the C3 exoenzyme. Therefore, in this study, we investigated the endocytotic uptake mechanism of C3 by use of different chemical inhibitors. Our results show that internalization of the C3 exoenzyme is dynamin-dependent, and the intermediate filament, vimentin, is involved in C3 binding and uptake.

## 2. Results

### 2.1. Cellular Susceptibility of HT22 and J774A.1 Cells towards the C3 Exoenzyme

**Figure 1:**
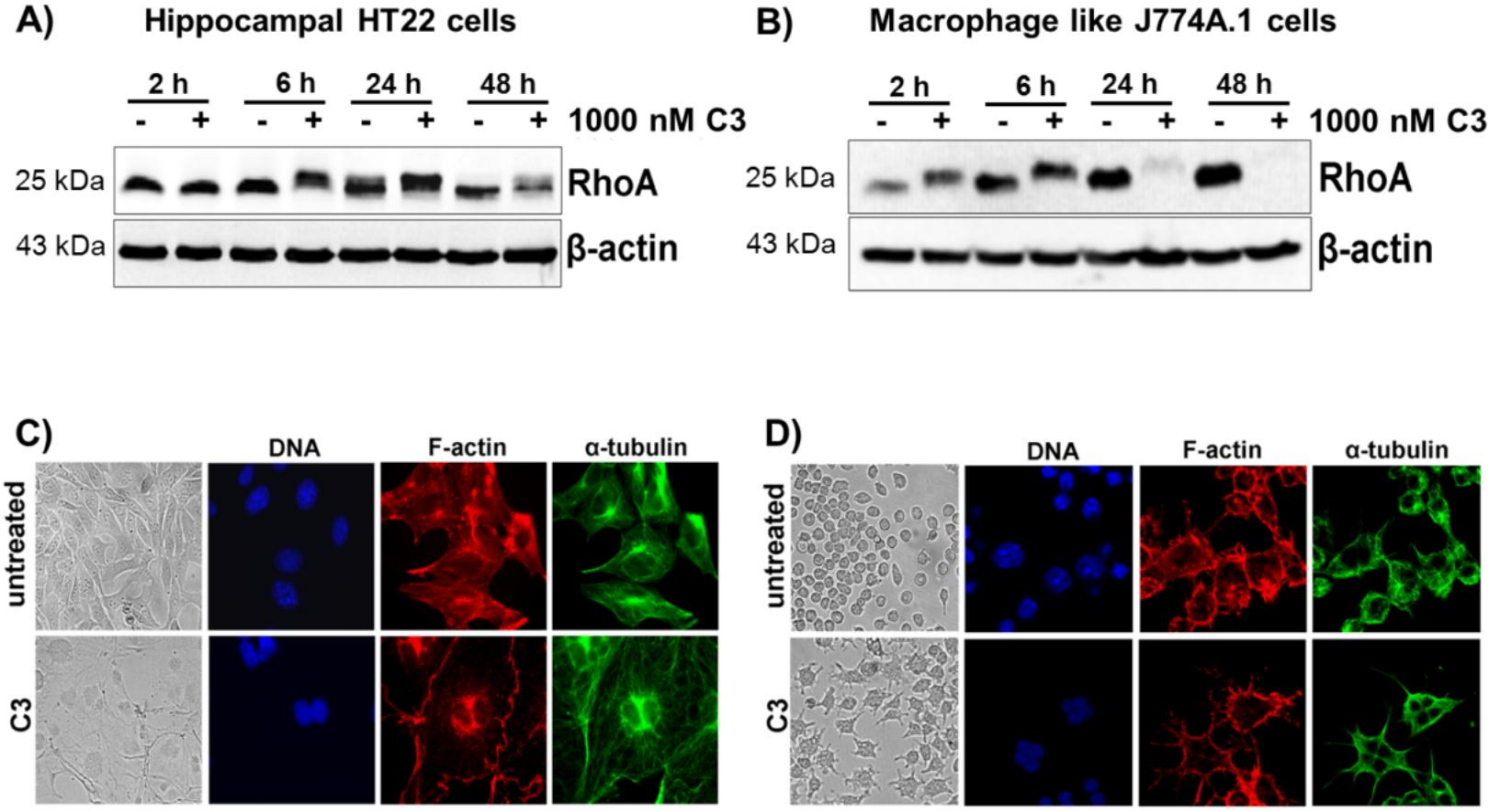
Cellular susceptibility of HT22 and J774A.1 cells to C3 hippocampal HT22. HT22 cells (**A**) and J774A.1 macrophages (**B**) were treated with 1 µM of C3 for the indicated time points at 37 °C. Cell lysates were submitted to western blot analysis probing RhoA and β-actin. One representative western blot experiment is shown (*n* = 3). C3-induced morphological changes of HT22 cells (**C**) and J774A.1 cells (**D**) were studied by phase-contrast microscopy and confocal laser scan microscopy after 48 h of incubation. For the immunofluorescence microscopy, the cells were fixed, permeabilized and stained for α-tubulin, F-actin and nuclei. Untreated cells served as the control.

C3-mediated ADP-ribosylation of RhoA results in an altered migration behavior of RhoA in SDS-PAGE. This gel-shift assay is used as the direct read out system for C3 uptake into cells. Therefore, western blots were used to detect ADP-ribosylated RhoA in cell lysates. We used the murine hippocampal HT22 cell line and J774A.1 mouse macrophages as model systems, because both cell lines were susceptible to C3 [7,11]. Treatment of HT22 cells with C3 caused a time-dependent ADP-ribosylation of RhoA starting at 6 h and finally resulting in the degradation of ADP-ribosylated RhoA after 48 h (Figure 1A). Due to the high sensitivity of J774A.1 cells towards C3, complete ADP-ribosylation of RhoA was detected within 2 h and almost complete degradation of ADP-ribosylated RhoA after 24 h (Figure 1B). Interestingly, pronounced morphological changes were not observed in both cell lines until 48 h. In both cell lines, C3 induced a multinucleated phenotype and an increase in cell size accompanied by the disappearance of actin stress fibers (Figure 1C,D). At this time point, only a few cells exhibited a rounded morphology with neurite-like extensions or bipolar protrusions. The RhoA shift, as well as the morphological changes confirmed the intracellular uptake and intracellular activity of C3. These results nicely confirm previous observations of our group and show that J774A.1 cells are more sensitive to C3 than the hippocampal HT22 cells.

### 2.2. Acidification of Endosomes is not Essential for the Uptake of C3

**Figure 2:**
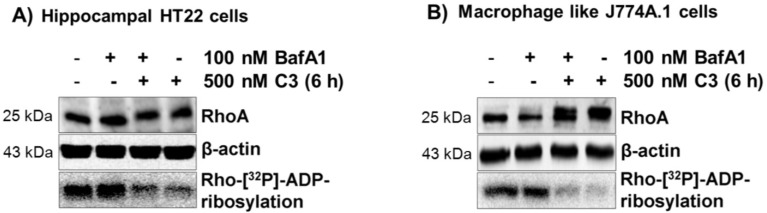
Uptake of C3 into HT22 and J774A.1 cells after inhibition of the vacuolar H(^+^)-ATPase on endocytosis by bafilomycin 1. HT22 cells (**A**) or J774A.1 cells (**B**) were incubated with 100 nM bafilomycin A1 for 1 h at 37 °C. Subsequently, 500 nM of C3 was applied, and the cells were further incubated at 37 °C in the presence of bafilomycin A1. In parallel, cells were incubated with C3 alone or left untreated. Cells were lysed and submitted to western blot analysis against RhoA and β-actin. The ADP-ribosylation state of Rho from cells treated with C3 in the absence or presence of bafilomycin A1 was determined by the ADP-ribosylation assay. Western blots and autoradiography from representative experiments are presented (*n* = 3).

To study whether C3 uptake requires an acidic cellular compartment for uptake, bafilomycin A1 was used, a specific inhibitor of the vacuolar ATPase-dependent proton pump [24]. As shown in Figure 2, bafilomycin A1 did not inhibit the uptake of C3 into HT22 cells (Figure 2A) nor into J774A.1 macrophages (Figure 2B), as intracellular ADP-ribosylation of RhoA was not influenced. Bafilomycin A1 was in fact effective at inhibiting endocytosis, as the uptake of the Rho glucosylating toxin B was strongly inhibited (Figure S1). *Clostridium difficile* toxin B enters the cells via acidic early endosomes [15].

**Figure 3:**
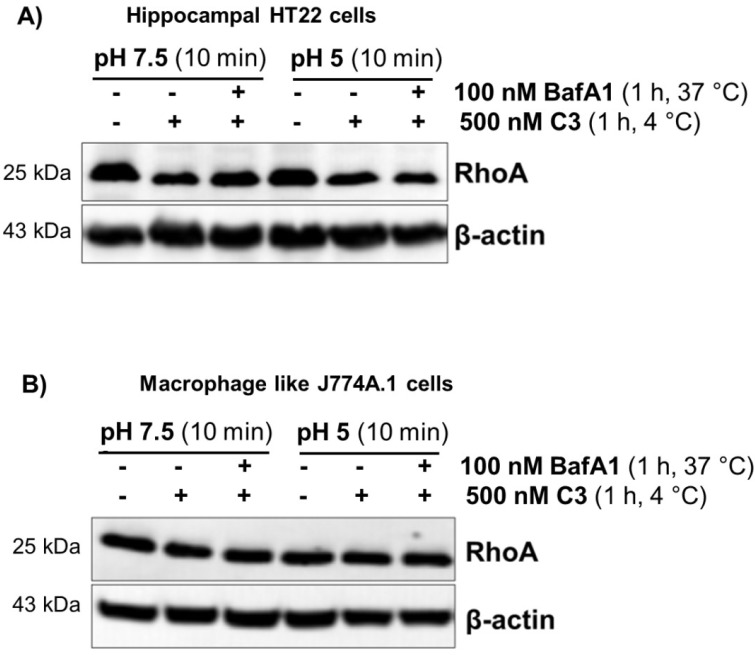
Uptake of C3 into HT22 and J774A.1 cells under extracellular acidic condition and in the presence of bafilomycin A1. Bafilomycin A1-pretreated HT22 cells (**A**) and J774A.1 cells (**B**) were incubated in serum-free medium with 500 nM C3 (or left untreated for control) at 4 °C. After 1 h, the medium was either adjusted to pH 5.0 or to pH 7.5, and cells were further incubated for 10 min, still in the presence of bafilomycin A1. Then, cells were incubated in neutral medium containing bafilomycin A1 at 37 °C for a further 6 h (HT22) and 4 h (J774A.1). Cells were lysed and submitted to western blot analysis probing RhoA and β-actin.

Bacterial protein toxins that are endocytosed via an acid compartment can be translocated through the plasma membrane by a short time drop in the extracellular pH value. This manipulation simulates the endogenous uptake process. To this end, C3 was bound to intact cells for 60 min at 4 °C, followed by a ten-minute drop to pH 5.0 at 37 °C, Then, the acidic medium was removed, and incubation was continued in medium (pH 7.5) at 37 °C (Figure 3). This experimental setting did not allow the uptake of C3, whereas toxin B was able to be directly translocated from the extracellular space into the cytoplasm (Figure S2). These results suggest that acidification of endosomes is not essential for the uptake of C3.

### 2.3. Cholesterol is not Involved in the Uptake of C3

**Figure 4:**
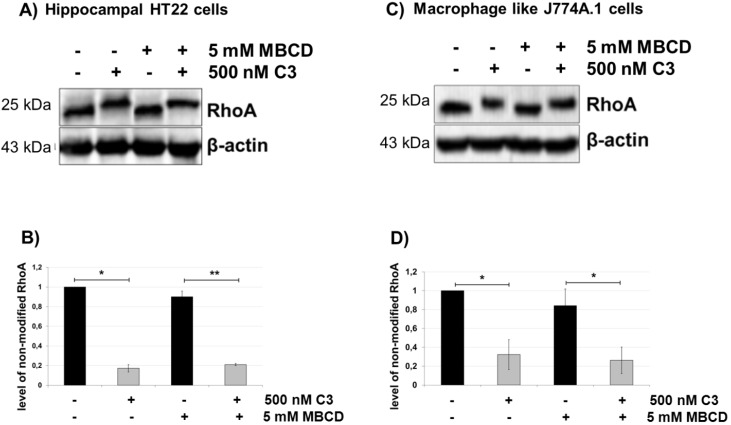
Uptake of C3 into HT22 and J774A.1 cells after cholesterol depletion by methyl-β-cyclodextrin (MBCD). Cultivated cells ((**A**) = HT22 cells, (**C**) = J774A.1 cells) were pre-treated with methyl-beta-cyclodextrin (5 mM) for 20 min followed by incubation with C3 (500 nM) for the indicated time. Cells were lysed and submitted to western blot analysis probing RhoA and β-actin. Densitometric evaluation of RhoA is shown ((**B**) = HT22 cells and (**D**) = J774A.1 cells). Non-shifted RhoA (indicative of non-modified Rho) is quantified by densitometric evaluation and adjusted to the corresponding β-actin signal. Results represent the arithmetic means ± SD of three independent experiments. Statistical differences between C3-treated and control cells were determined using a two-sided Student’s *t* test (* *p* ≤ 0.05; ** *p* ≤ 0.01).

To assess the involvement of cholesterol-containing membranes in the uptake of C3, HT22 cells and J774A.1 macrophages were treated with the cholesterol-depleting agent, methyl-β-cyclodextrin (MBCD), for 20 min followed by C3 incubation for the indicated time. No differences in ADP-ribosylation of RhoA were found between MBCD-treated cells and cells that were subjected only with C3 without MBCD pre-treatment (Figure 4). The reduction of pore formation of *Clostridium difficile* toxin A was already detectable at concentrations of 0.5 mM MBCD. Moreover, 2.5 mM and 5 mM MBCD resulted in complete inhibition of membrane permeabilization and significantly reduced the intoxication of cells by toxin A and toxin B [25]. The used concentration of MBCD was effective t inhibiting endocytosis, as the uptake of the Rho glucosylating toxin B was strongly inhibited (Figure S3A). However, cholesterol depletion of HT22 cells by treatment with 10 mM of MBCD for 20 min was cytotoxic and induced apoptosis (Figure S3B). Additionally, the observed results were consistent with those obtained with nystatin and filipin treatment of HT22 and J774A.1 cells. In these experiments, the pre-treatment of cells with cholesterol-complexing agents did not inhibit ADP-ribosylation of RhoA and, thus, did not inhibit uptake of C3. These results indicated that cholesterol and lipid rafts are not involved in the uptake mechanism of C3.

### 2.4. Intermediate Filaments are Involved in the Uptake of C3

**Figure 5:**
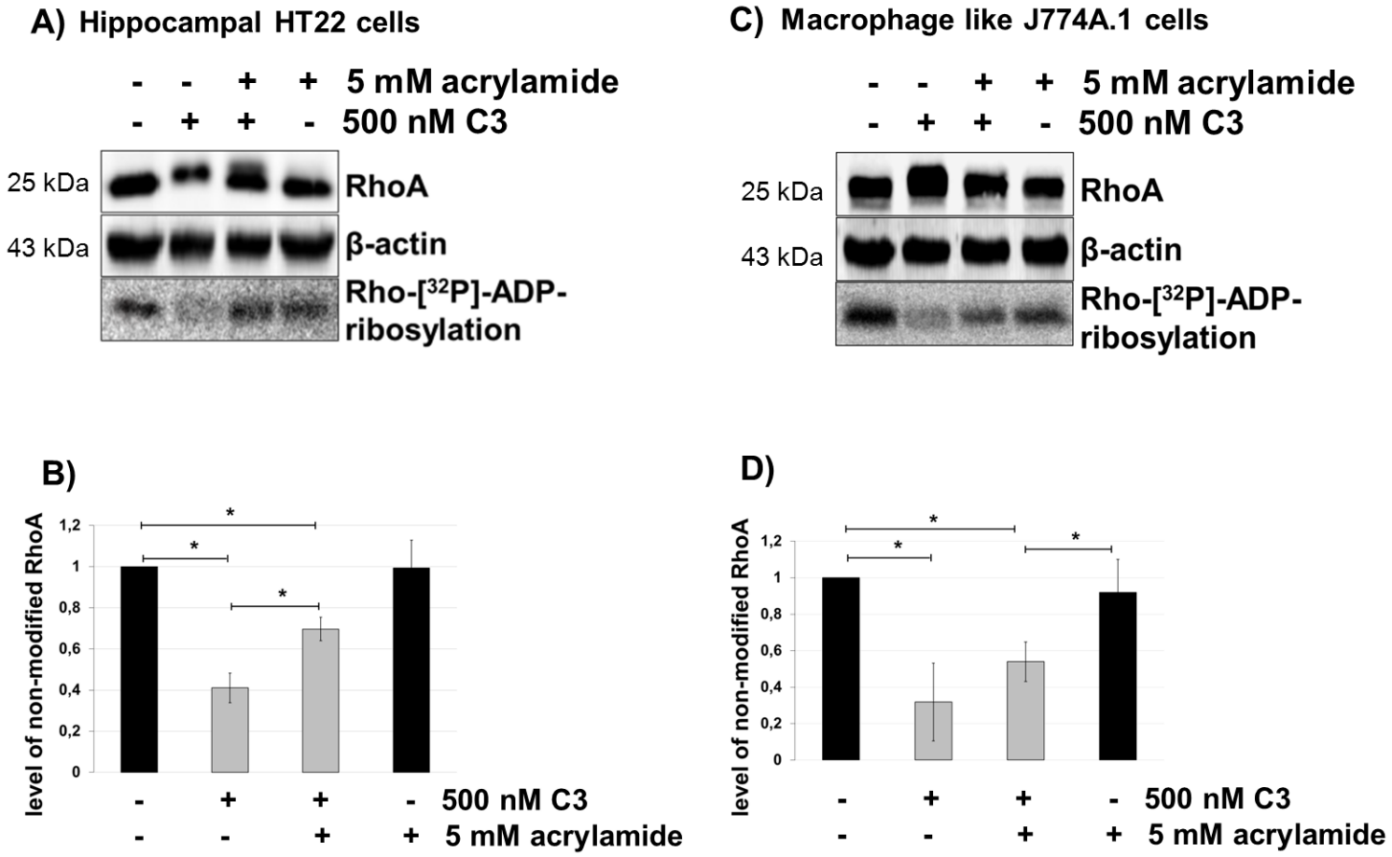
Uptake of C3 into HT22 and J774A.1 cells after disruption of the vimentin network by acrylamide treatment. Cultivated cells ((**A**) = HT22 cells, (**C**) = J774A.1 cells) were pre-treated with acrylamide (5 mM) for 30 min followed by incubation with C3 (500 nM) for the indicated time. Cells were lysed and submitted to western blot analysis probing RhoA and β-actin and the ADP-ribosylation assay. After C3 exoenzyme treatment with or without acrylamide, the cells were lysed, and lysate proteins (4 µg) were subjected to an *in vitro* [^32^P]-ADP-ribosylation assay with C3. [^32^P]-ADP-ribosylated RhoA is quantified densitometrically and normalized to the control level of non-modified RhoA ((**B**) = HT22 cells, (**D**) = J774A.1 cells). Statistical differences between C3-treated and control cells were determined using a two-sided Student’s *t* test (* *p* ≤ 0.05).

Acrylamide, a known selective disruptor of intermediate filaments [26], leaving microtubuli and actin networks intact [27,28], was used to study a possible involvement of vimentin in C3 uptake. Five millimolar acrylamide strongly decreased the uptake of C3 shown in the gel-shift and ADP-ribosylation assay (Figure 5A,B for HT22 cells and C,D for J774A.1 cells). This result was confirmed by the observation that acrylamide pre-treatment of J774A.1 cells caused fewer cells with C3-induced bipolar protrusions. In addition, the neurite-like protrusions were also significantly shorter as compared with C3-treated cells. These results indicate that an intermediate filament, such as vimentin, is involved in the uptake of C3. Microtubules and actin filaments, however, were not involved, as the nocodazole and latrunculin B treatment of cells had no effect on ADP-ribosylation of RhoA.

### 2.5. Dynasore Inhibits the Uptake of C3

To determine the involvement of classical endocytosis in the uptake of C3, HT22 cells were pre-treated with chlorpromazine (5 µg/mL), which selectively inhibits clathrin-dependent endocytosis and brefeldin A (10 µM), which disrupts trafficking between the Golgi apparatus and the endoplasmic reticulum (ER) by inhibition of the small GTPase, ARF [29]. No difference in ADP-ribosylation of RhoA between C3 and chlorpromazine plus C3-treated cells or between C3 and brefeldin A in combination with C3 was detected, indicating that the classic clathrin-endocytotic pathway was not involved in cell entry of C3.

**Figure 6:**
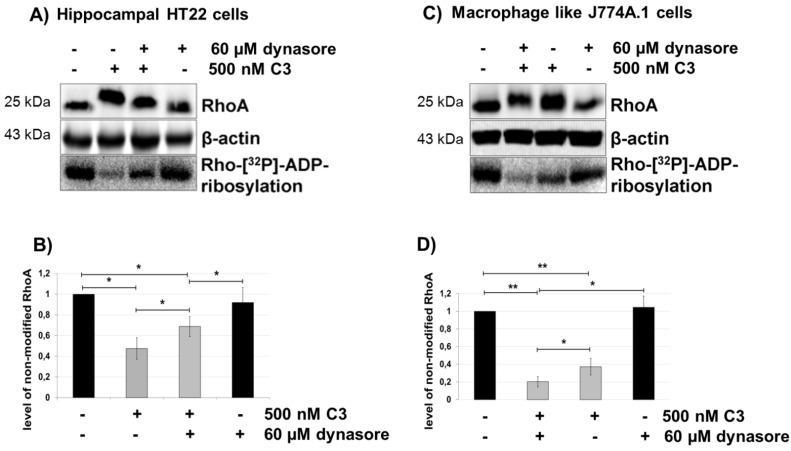
Uptake of C3 into HT22 and J774A.1 cells after inhibition of endocytosis by dynasore treatment. HT22 cells (**A**) and J774A.1 macrophages (**C**) were pre-treated with dynasore (60 µM) for 45 min followed by incubation with C3 (500 nM) for the indicated time. Cells were lysed and subjected to western blot analysis using antibodies against RhoA or β-actin. The ADP-ribosylation state of Rho from cells treated with C3 in the absence or presence of dynasore was determined by the ADP-ribosylation assay. After C3 exoenzyme treatment with or without dynasore, the cells were lysed, and lysate proteins (4 µg) were subjected to an *in vitro* [^32^P]-ADP-ribosylation assay with C3. [^32^P]-ADP-ribosylated RhoA is quantified densitometrically and normalized to the control level of non-modified RhoA ((**B**) = HT22 cells, (**D**) = J774A.1 cells). Statistical differences between C3-treated and control cells or C3-treated alone or in combination with dynasore were determined using a two-sided Student’s *t* test (* *p* ≤ 0.05; ** *p* ≤ 0.01).

Dynasore is a non-competitive inhibitor of the GTPase activity of dynamin and blocks dynamin-dependent endocytosis in cells, including hippocampal neurons [30,31]. Prior to the addition of C3, cells were incubated with 60 µM of dynasore or 0.16% of DMSO only (vehicle control) in DMEM for 45 min at 37 °C. As shown in Figure 6A,B, dynasore (60 µM) resulted in strong inhibition of the internalization of C3, as evidenced by diminished RhoA-shift in HT22. Extended treatment of HT22 (up to 14 h) cells with 60 μM of dynasore resulted in slight RhoA degradation. In contrast, dynasore-treated J774A.1 macrophages showed enhanced ADP-ribosylation of RhoA, as determined by the complete mol weight shift of RhoA and degradation of inactivated RhoA (Figure 6C,D). Despite these controversial results, the participation of dynamin in the C3 uptake was demonstrated in both cell lines.

## 3. Discussion

Little is known about the mode of internalization of C3 exoenzyme into cells. Nonspecific pinocytosis was proposed as a mechanism of entry, as high concentrations of C3 and prolonged incubation times are apparently needed [32]. A preliminary study indicates that *Staphylococcus aureus* invades target cells and releases a C3-like exoenzyme into the cytosol [33]. Only one study reported a selective uptake by macrophages through acidified early endosomes [34].

**Figure 7:**
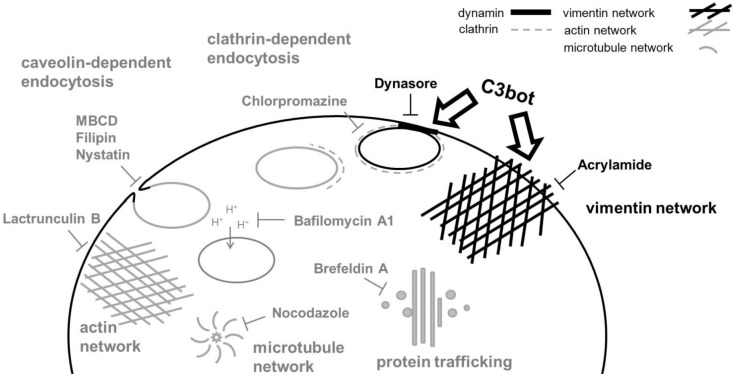
General cellular uptake mechanisms and their inhibition by drugs.

We investigated the mechanism of C3 uptake using different pharmacological inhibitors of endocytosis and presented data that C3 exoenzyme is endocytosed by a mechanism that does not depend strictly on the classic endocytotic pathway (Table 1 and Figure 7).

**Table 1:** Effects of C3 uptake of the tested compounds. (−, no effect on the RhoA shift; +, RhoA shift is delayed/faster or missing compared to the control).

Compound	Mechanism	HT22	J774A.1
Bafilomycin A1	blocks endosomal acidification	−	−
Brefeldin A	blocks protein trafficking	−	−
Methyl-β-cyclodextrin (MBCD)	blocks caveolin-dependent endocytosis	−	−
Filipin	blocks caveolin-dependent endocytosis	−	−
Nystatin	blocks caveolin-dependent endocytosis	−	−
Chlorpromazine	blocks clathrin-dependent endocytosis	−	−
Latrunculin B	blocks actin polymerization	−	−
Nocodazole	blocks polymerization of microtubules	−	−
Acrylamide	interrupts vimentin filament network	+	+
Dynasore	blocks dynamin-dependent endocytosis	+	+

Several routes of endocytic uptake do exist in cells. The best characterized and intensively studied pathway is clathrin-mediated endocytosis [35]. In addition, clathrin-independent endocytotic pathways have been reported. Clathrin-independent endocytosis comprises multiple different mechanisms, involving, for example, caveolins, dynamin, interleukin receptor 2β, flotillin, Cdc42 or Arf6 [20,36].

By regulating the cellular pH balance, intracellular vacuolar H^+^ ATPases are important for intracellular trafficking, protein processing, translocation, degradation and the coupled transport of small molecules and ions into cells [37,38,39]. Vacuolar H^+^ ATPase inhibitor bafilomycin A1 is a potential inhibitor of endocytosis by inhibiting the acidification of organelles, such as endosomes and lysosomes [40,41]. Bafilomycin A1 did not prevent the release of C3 into the cytosol of the studied cells, neuronal HT22 cells, as well as J774A.1 macrophages. These results were supported by the observation that extracellular acidification (mimicking the endosomal process) did not enhance the uptake of C3 into cells. The data indicate that C3 does not require an acidic compartment for uptake. Our data are not in agreement with the recently published study reporting that acidic endosomes are partially involved in the uptake and translocation of C3 into J774A.1 cells [34]. In that study, a bafilomycin A1-mediated partial inhibition of C3 uptake into J774A.1 macrophages and human promyelocytic leukemia HL-60 cells was shown. Surprisingly, the effect seems to depend on the C3 concentration. At a high C3 concentration (2 µg/mL = 85 nM), bafilomycin A1 has almost no effect on C3 uptake. In contrast, we did not detect any effect of bafilomycin, even at a low C3 concentration (0.24 µg/mL = 10 nM). This discrepancy is surprising and can only be explained be the differences in cell sub-clones.

The majority of endocytic mechanisms depend on free cholesterol in the membranes [42,43]. This form of endocytosis is often mediated by caveolae, which are non-clathrin-coated membrane invaginations at the cell surface [44]. Based on their lipid composition, caveolae are considered to be subtypes of lipid rafts [45] and are capable of mediating endocytosis [46]. Caveolae flatten or disappear in cells that are depleted of cholesterol [45] and cause the inhibition of endocytosis. Accordingly, treatment with methyl-beta-cyclodextrin (MBCD), a cholesterol trapping agent, should result in reduced C3 uptake. However, we found that methyl-beta-cyclodextrin did not inhibit the uptake of the C3 exoenzyme into HT22 and J774A.1. This result was supported by the finding that neither nystatin nor filipin treatment protect cells from C3 entry. In addition, C3 uptake was not affected by chlorpromazine, which is a known inhibitor of clathrin-dependent endocytosis [47].

By contrast, acrylamide disrupts the organization of the intermediate filament vimentin networks [27]. Binding of C3 depends on vimentin [48]. Therefore, the uptake of C3 in acrylamide pretreated cells was examined. In fact, acrylamide caused a decrease in C3 uptake determined by gel-shift and RhoA ADP-ribosylation assays. It is conceivable that acrylamide alters vimentin-driven endo-lysosomal vesicle transport [49] and endocytotic uptake [50,51]. Recently, Bonfiglio and co-workers reported that vimentin translocates to the membranes of HT22 cells and associates with dynamin to be in charge of the internalization of the cortico-releasing hormone receptor-β-arrestin 2 complex [52]. Consistent with these observations, we demonstrate that functional inhibition of dynamin by dynasore results in a diminished uptake of C3 into neuronal HT22 cells. Unexplainably, the uptake of a C3 in macrophage-like J774A.1 cells was slightly upregulated upon dynasore treatment. This result is in agreement with earlier reports showing that treatment of J774 cells with 80 μM of dynasore resulted in upregulation in endocytosis of transferrin-AF647, internalized mainly by dynamin- and clathrin-mediated endocytosis. Moreover, dynasore at a concentration of 40–80 μM did upregulate the internalization of lipopolysaccharide (LPS) (1000 ng/mL) by about 20%–30% [53]. This discrepancy is probably based on differences in motility. Macrophages are highly motile cells. These cells change their cell shape very quickly and, therefore, their cytoskeleton and focal adhesion complexes. These dynamic processes are accompanied by an increased endocytotic mechanism, so that proteins can be delivered to the cell periphery. This mechanism is strictly regulated and certainly different in non-motile cells, like HT22 cells.

However, although the C3 uptake was clearly reduced by acrylamide and dynasore treatment, it could not completely block the entry of C3 into cells. Therefore, C3 likely enters cells via an additional uptake-pathway. Indeed, bacterial toxins recruit more than one uptake pathway, as for example described for Shiga toxin [54] or for cholera toxin [55]. Shiga toxin, which binds to the glycolipid receptor (Gb3) [56], can be efficiently endocytosed by clathrin-coated pits [57] and clathrin-independent endocytosis [58].

In summary, we demonstrated that internalization of the C3 exoenzyme occurs via an intermediate filament- and dynamin-dependent mechanism and that this pathway of endocytosis participates in C3 uptake, but it is not the only one that does.

## 4. Experimental Section

### 4.1. Cell Culture

Murine hippocampal HT22 cells were cultivated in Dulbecco’s modified essential medium (Biochrom, +10% FCS, 1% penicillin, 100 units/mL streptomycin and 1 mM sodium pyruvate). J774A.1 mouse macrophages were cultivated in RPMI 1640 Medium (Biochrom; with 10% FCS, 1% penicillin, 100 units/mL streptomycin and 1 mM sodium pyruvate). Cells were maintained at 37 °C and 5% CO_2_. Upon subconfluence, cells were passaged.

Cholesterol depletion, which affects caveolae-mediated endocytosis, was carried out by pre-incubating cells in 5 mM methyl-β-cyclodextrin (Sigma-Aldrich Chemie GmbH, Munich, Germany) for 20 min at 37 °C followed by incubation with 500 nM C3 for 6–24 h.

Bafilomycin A1 (Sigma-Aldrich Chemie GmbH, Munich, Germany) was used to block the endosomal acidification. HT22 or J774A.1 cells were pretreated with 100 nM bafilomycin A1 and then incubated with 500 nM C3 for 6 h at 37 °C. To test whether extracellular acidic conditions influence the uptake of C3, cultivated cells were preincubated for 30 min at 37 °C with bafilomycin A1. Thereafter, the medium at pH 7.5 or 5.0 containing bafilomycin plus the C3 exoenzyme was added to the cells. After incubation of the cells for 10 min at 37 °C, fresh medium (37 °C, pH 7.5) containing bafilomycin A1 was added, and cells were further incubated for 4 or 6 h at 37 °C.

Dynamin inhibitor dynasore was from Sigma (Sigma-Aldrich Chemie GmbH, Munich, Germany). Cells were pretreated with 60 µM dynasore for 45 min, followed by incubation with 500 nM C3 for 14 h. Acrylamide (Sigma-Aldrich Chemie GmbH, Munich, Germany) was used to disrupt the intracellular vimentin network. Five millimolar acrylamide was applied to HT22 or J774A.1 cells for 1 h, followed by incubation with 500 nM C3 for the indicated time.

Further commercially-obtained compounds—brefeldin A (10 µM), nystatin (100 µg/mL) and filipin (3 µg/mL)—were from Sigma (Sigma-Aldrich Chemie GmbH, Munich, Germany), and chlorpromazine (5 mg/mL), nocodazole (20 µM) and latrunculin B (4 µg/mL) were from Merck (Merck Millipore KGaA, Darmstadt, Germany). For inhibitory experiments, serum-free medium was used.

The morphological changes of treated cells were analyzed by phase-contrast microscopy using a Zeiss Axiovert 200 M (Zeiss Axioplan, Zeiss, Oberkochem, Germany).

### 4.2. Expression and Purification of Recombinant C3 Protein

C3bot from *Clostridium botulinum* (Accession No. X59039) was expressed as recombinant GST-fusion protein in *E. coli* TG1 harboring the respective DNA fragment in the plasmid, pGEX-2T, and purified by affinity chromatography using glutathione-sepharose. The fusion protein was eluted from the beads using glutathione [7].

### 4.3. Western Blot Analysis

Complete lysate proteins were separated using sodium dodecylsulfate polyacrylamide gel electrophoresis (SDS-PAGE) (Cti-Chemie u. Werkstoff-Technik GmbH, Idstein, Germany) and subsequently transferred onto nitrocellulose membranes by a tank blot system. The membranes were blocked with 5% (*w/v*) nonfat dried milk for 60 min; incubation with primary antibody was conducted overnight at 4 °C and treatment with the secondary antibody at room temperature for 1 h. For western blot analysis, the following primary antibodies were used: RhoA was identified using a mouse monoclonal IgG from Santa Cruz Biotechnologies (Santa Cruz, CA, USA). Identification of C3 was achieved by a rabbit polyclonal antibody (affinity purified), which was raised against the full-length toxin, C3bot (Accession No. CAA41767). Actin (Sigma-Aldrich Chemie GmbH, Munich, Germany) was used as the loading control. For the chemiluminescence reaction, electrochemiluminescence (ECL) Femto (Pierce, Thermo Fisher Scientific Inc., Rockford, IL, USA) was used. All signals were analyzed densitometrically using the KODAK 1D software (KODAK GmbH, Stuttgart, Germany) and normalized to β-actin signals.

### 4.4. Immunocytochemistry

Cells seeded on cover slips were washed with PBS and subsequently fixed in 4% formaldehyde in phosphate-buffered saline (PBS) (pH 7.4) at room temperature for 15 min. Cells were then washed and permeabilized with 0.3% (*w/v*) Triton X-100 in PBS supplemented with 5% BSA. Tubulin was stained by α-tubulin antibody (YL 1/2, Linaris Biologische Produkte GmbH, Dossenheim, Germany) and Alexa 488-conjugated secondary antibody (Life Technologies GmbH, Darmstadt, Germany) for 1 h at room temperature. Actin staining was performed using rhodamine-conjugated phalloidin (Sigma-Aldrich Chemie GmbH, Munich, Germany) for 30 min at room temperature. Then, a 0.1 µg/mL solution of DAPI (Serva Electrophoresis GmbH, Heidelberg, Germany) in phosphate-buffered saline supplemented with 0.1% (*w/v*) Tween-20 was used for nuclei staining for 15 min at 37 °C. Cells were analyzed by confocal laser scanning microscopy using a Leica TCS SL microscope (Leica Mikrosysteme Vertrieb GmbH, Wetzlar, Germany).

### 4.5. Confocal Laser Scanning Microscopy

For image acquisition, a Leica TCS SL confocal laser scanning microscope (Leica Mikrosysteme Vertrieb GmbH, Wetzlar, Germany) using a 63× oil immersion objective was used. Fluorescent dyes were excited at a wavelength of 488 nm (green fluorescence), 543 nm (red fluorescence) and 405 nm (blue fluorescence), respectively. Images were captured at a resolution of 1024 × 1024 pixels.

### 4.6. ADP-Ribosylation of Rho in Murine Cells

To verify the effectiveness of ADP-ribosylation of Rho by *C. botulinum* exoenzyme C3, the cultivated cells were either left untreated or were incubated with 500 nM C3 for 6 h. The cells were then washed with NaCl/Pi and scraped into 100 µL of lysis buffer (20 mM Tris/HCl (pH 7.4), 1% Triton X-100, 10 mM NaCl, 5 mM MgCl2, 1 mM phenylmethanesulfonyl fluoride, 5 mM dithiothreitol). The obtained suspension was shaken at 37 °C for 10 min. Ultrasonic disruption was performed using a cycle of 10× 5 s, 5× 10% sonic energy with a sonotrode (Bandelin Electronic). Protein concentrations were measured by the Bradford method. Cell lysates containing equal amounts of protein were incubated with 1000 nM recombinant *C. botulinum* exoenzyme C3 and 1 µCi [^32^P]NAD (Amersham Life Sciences, Arlington Heights, IL, USA) in 20 µL of 4× buffer containing 50 mM HEPES (pH 7.3), 10 mM MgCl_2_, 10 mM dithiothreitol, 10 mM thymidine and 10 µM NAD at 37 °C for 20 min. The reaction was terminated by the addition of Laemmli sample buffer and then incubated at 95 °C for 10 min. Samples were resolved by SDS/PAGE on 15% gels, and the ADP-ribosylated Rho was analyzed by phosphorimaging (Cyclone, Packard American Instrument, Haverhill, MA, USA).

### 4.7. Reproducibility of the Experiments and Statistics

All experiments were performed independently at least three times. Results from representative experiments are shown in the figures.

## Supplementary Materials

Supplementary materials can be accessed at: http://www.mdpi.com/2072-6651/7/2/0380/s1.
